# The Effect of μ-Limestone Additions on the Mechano-Chemical and Microstructural Properties of Slag and Binary Slag/Ground Fly Ash Alkaline-Activated Binders

**DOI:** 10.3390/ma17235940

**Published:** 2024-12-04

**Authors:** Francisco Javier Vázquez-Rodríguez, Lucio Guillermo López-Yépez, Nora Elizondo-Villarreal, Ana María Guzmán-Hernández, Lauren Yolanda Gómez-Zamorano, Edén Amaral Rodríguez-Castellanos

**Affiliations:** 1 Programa Doctoral en Ingeniería Física, Facultad de Ciencias Físico Matemáticas, Universidad Autónoma de Nuevo León, Av. Pedro de Alba s/n, Ciudad Universitaria, San Nicolás de los Garza C.P. 66451, NL, Mexico; fvazquezr@uanl.edu.mx (F.J.V.-R.); nora.elizondovl@uanl.edu.mx (N.E.-V.); 2 Facultad de Arquitectura, Universidad Autónoma de Nuevo León, Av. Pedro de Alba s/n, Ciudad Universitaria, San Nicolás de los Garza C.P. 66451, NL, Mexico; 3 Facultad de Ingeniería Civil, Universidad Autónoma de Nuevo León, Ciudad Universitaria, San Nicolás de los Garza C.P. 66455, NL, Mexico; llopezy@uanl.edu.mx; 4 Facultad de Ingeniería Mecánica y Eléctrica, Universidad Autónoma de Nuevo León, Av. Pedro de Alba s/n, Ciudad Universitaria, San Nicolás de los Garza C.P. 66450, NL, Mexico; ana.guzmanhr@uanl.edu.mx

**Keywords:** slag, fly ash, limestone, alkali-activated cement, hybrid gels, Fourier-transform infrared spectroscopy (FTIR), X-ray diffraction (XRD), scanning electron microscopy (SEM)

## Abstract

An alternative approach to reducing the clinker factor, i.e., worldwide CO_2_ emissions resulting from the production of composite cement, is to replace these materials with supplementary aluminosilicate-based materials that promote the formation of alkali-activated cements, whose elevated temperature resistance, limited permeability, strong binding properties, excellent durability, high chemical corrosion resistance, confinement of toxic waste, and environmentally low impact have attracted a lot of attention in the cement industry. The principal aluminosilicate-based supplementary materials (SCMs) used in the cement industry are fly ash and blast-furnace slag. Recently, limestone has been proposed for use in alkali-activated cement to improve mechanical resistance and promote nucleation sources for the hydration of hybrid gels. In the current research work, the effect of 5 and 10 wt% limestone additions to slag and fly ash/slag alkali-activated cements with NaOH-4M was studied to evaluate the mechano-chemical and microstructural properties of alkali-activated cement. The effect of limestone was studied using mechanical resistance, XRD, FTIR, SEM-EDS, and calorimetry methods. The XRD, FTIR, and SEM-EDS results demonstrated the formation of portlandite Ca(OH)_2_ after the activator solution’s reaction with limestone. The limestone’s dissolution in Ca^2+^ contributes to hybrid gel formation ((N, C)-A-S-H, N-A-S-H, and C-A-S-H), resulting in compressive strength higher than 20 MPa, the recommended resistance for commercial cement.

## 1. Introduction

Blended cements are cements in which a proportion of the Portland cement clinker is replaced by industrial by-products, such as granulated blast-furnace slag (GBS), power station fly ash (also known as PFA), some types of volcanic material (natural pozzolanas), or limestone. Blended cement formulations can include the following combinations: (i) clinker + fly ash + blast-furnace slag, (ii) clinker + fly ash + limestone powder, or (iii) clinker + blast-furnace slag + limestone. The typical range of blended cement components is 35% to 65% clinker, 15% to 35% fly ash, 20% to 50% granulated slag, and 3% to 5% gypsum. Multi-component cements offer significant benefits, including a 30–40% reduction in fuel energy consumption and concrete production volumes. However, in the long term, CO_2_ emissions may incur penalties. As a result, clinker will gradually be substituted with materials such as fly ash, slag, limestone powder, and natural pozzolans. Global projections for Portland cement demand are estimated to reach approximately 6 billion tons annually, generating over 4.8 billion tons of CO_2_ emissions annually. CO_2_ is known as the main greenhouse gas (GHG) responsible for global warming. The cement industry contributes ~8% of CO_2_ emissions worldwide, causing serious alarm [1]. A feasible alternative to reduce the clinker factor in the production of blended cement is to replace Portland cement with alkali-activated cement, using supplementary aluminosilicate-based materials activated by alkaline mediums. It is well established that the alkaline activation of powders derived from industrial by-products containing aluminosilicate materials, commonly known as precursors, can produce inorganic binders due to the reaction between alkaline compounds and precursors. The formation of these inorganic binders comes from alkali activation. Alkali activations have gained interest because they produce materials with elevated temperature resistance, limited permeability, strong linkage, excellent durability, high chemical corrosion resistance, the ability to immobilize of toxic waste, and that are environmentally friendly. The raw materials used in alkali-activated cement synthesis often originate from industrial aluminosilicate waste, natural aluminosilicate minerals, or a combination of both [2,3].

It has been noted that substantial amounts of ground granulated blast-furnace slag (GGBFS) and fly ash (FA) remain accessible globally. This fact leads to the opportunity to investigate new alkali-activated cement development through the use of GGBFS and FA due to their high hydraulic potential and economic and environmental advantages [4,5,6,7].

Class F fly ash (containing less than 20% CaO) is a prime material for alkali-activated cement synthesis owing to its affordability, widespread availability, favorable spherical morphology, and abundant reactive aluminates and amorphous silicates. A few years ago, its annual production was estimated to be around 900 million tons [8]. During the alkali activation of fly ash, Si^4+^ or Al^3+^ ions react with Ca^2+^ from either the fly ash itself or added calcium-containing substances to produce calcium–silicate–hydrate gel (C-S-H), calcium–aluminate–hydrate gel (C-A-H), or calcium–aluminum–silicate–hydrate gel (C-A-S-H) in the presence of water [9,10]. Ca^2+^ plays a crucial role in accelerating the nucleation and aggregation of C-A-S-H and C-S-H gels [11]. The rapid formation of amorphous C-A-S-H and C-S-H gels leads to a reduced setting time and decreased porosity in the final product. However, this quick setting can hinder the development of hydration gel (N-A-S-H). Increasing the NaOH concentration can extend the setting time by limiting calcium leaching, thus allowing the alkali activation process to proceed more gradually and to better control the paste’s setting behavior [12]. The alkali activation of fly ash with high hydroxide concentrations produces N-A-S-H gel that can reach strengths exceeding 60 MPa when cured under heat.

Alternatively, ground granulated blast-furnace slag (GGBFS) is a precursor material in alkali-activated cement synthesis and has been widely studied [13,14,15]. Its annual production is approximately 400 million tons. GGBFS is another suitable by-product material that can be used as a precursor for alkali-activated cements due to its glassy phase and because it is rich in amorphous calcium, silica, and alumina [16]. The primary reaction product of GGBFS is a C-A-S-H gel, which enhances the setting time and strength properties of geopolymers [13,14,15,17,18,19,20,21,22].

It has been observed that C-A-S-H and N-A-S-H gels can exist simultaneously when slag/ash composites are blended. Microstructural features reveal a significant influence of calcium on the chemical gel, particularly in environments where chemically bound water is present within the gel. A hybrid gel, (N, C)-A-S-H, has also been recognized within slag systems, stemming from the calcium released during slag dissolution and its integration into the N-A-S-H-type gel produced through ash activation [23,24].

Idawati et al. examined geopolymer composites derived from FA/GBFS, concluding that the geopolymerization of the GBFS-based formulation was governed by the C-A-S-H-type gel. In contrast, the FA-based geopolymer was impacted by sodium aluminosilicate (N-A-S-H)-type gel [25]. Kumar et al. substituted 5–50% of FA with GBFS to create a geopolymer at a temperature of 27 °C, discovering that the reaction was regulated by the dissolution and precipitation of the C-S-H-type gel [26].

Wang et al. assessed geopolymer formulations based on FA/GBFS with an FA/GBFS ratio ranging from 20 to 60 wt%, utilizing three concentrations of NaOH solutions (0.5%, 1%, and 1.5%) and curing the mixtures for 1, 3, 7, and 28 days. As slag concentration increased, so did the compressive strength of the geopolymer, reaching about 93 MPa [11]. Deb et al. activated GBFS/FA-based geopolymers with GBFS/FA ratios of 0%, 10%, and 20%, using NaOH and Na_2_SiO_3_ solutions, respectively. The compressive strength increased at higher GBFS/FA ratios.

Conversely, numerous studies have shown that limestone (CaCO_3_) incorporation enhances the properties of Portland cement. CaCO_3_ enhances early strength development due to the nucleation effect, accelerating the hydration process. Additionally, limestone incorporation improves workability, reducing the water demand for a given consistency, which is especially beneficial in concrete mixtures. It can also enhance durability by refining the pore structure and reducing permeability. Therefore, it increases resistance to aggressive environmental factors such as chlorides and sulfates. Finally, using limestone can lower the carbon footprint of cement production by reducing clinker content without significantly impacting performance, aligning with sustainability goals.

The incorporation of minerals with high Ca^2+^ content promotes the formation of hydration gels (C-A-S-H) that contribute to (i) improving the microstructural packing that provides network strength and (ii) enhancing the workability of pastes [27,28,29,30]. Fine limestone additions regulate setting time in high-fly-ash cement. Also, it has been reported that a 5% volume substitution of nano-limestone accelerates reactions and reduces setting time [31]. Some researchers have shown that CaCO_3_ is not inert; it reacts with Portland cement phases or fly ash compounds at early ages, enhancing mechanical and durability properties [32,33,34,35,36].

According to the literature, the reactivity of limestone under alkaline conditions plays a crucial role in modifying the properties of geopolymer and alkali-activated systems. Limestone, primarily composed of calcium carbonate (CaCO_3_), is relatively inert in neutral or mildly acidic environments. However, under the highly alkaline conditions (pH > 12) typically present in alkali-activated systems, it can partially dissolve, releasing calcium ions (Ca^2+^) into the solution. This dissolution is a key aspect of its reactivity, since these ions interact with silicate (SiO_4_^4−^) and aluminate (AlO_4_^5−^) species, leading to the formation of secondary hydration products that strengthen and densify the binder matrix, improving mechanical properties and durability [37]. The dissolved Ca^2+^ ions can react with silicate species in the solution to form calcium–silicate–hydrate (C-S-H) or calcium–aluminosilicate–hydrate (C-A-S-H) phases. These phases are essential for strength development and durability in alkali-activated cements, as they contribute to additional binding within the matrix. While C-S-H is more commonly found in Portland cement, the formation of C-A-S-H in alkali-activated systems can be promoted by reactive aluminosilicate precursors. These secondary gels coexist with the primary aluminosilicate network, enhancing the structural integrity of the matrix [38]. C-S-H and C-A-S-H gels promote a denser matrix to increase compressive strength, a critical factor for structural applications [39]. Furthermore, the presence of CaCO_3_ from limestone also imparts improved carbonation resistance due to the denser microstructure formed by secondary phases. This densification is beneficial for long-term durability, especially in environments exposed to CO_2_, as the carbonate content buffers the pH, potentially reducing the carbonation rate of the system [40].

On the other hand, adding limestone has several effects on alkali-activated cement (AAC) properties due to its ability to alter the matrix structure, provide nucleation sites, and moderate the system’s reactivity. Limestone can act as a filler, enhancing the microstructure by filling voids and creating a denser matrix. This filler effect enhances early-age compressive strength by improving particle packing and providing additional nucleation sites, accelerating the reaction rate and promoting faster strength development [37]. By promoting the formation of secondary gels, limestone addition improves the resistance of alkali-activated cements to leaching and chemical attack. This stabilizes the matrix and reduces permeability, enhancing durability in aggressive environments [41,42]. Lime accelerates reaction rates within AACs, effectively reducing setting times. Faster setting is beneficial in industrial applications where curing times impact production costs and time efficiency [43]. Limestone can act as a pH buffer, stabilizing the alkaline environment and preventing excessive alkalinity, which may lead to alkali–aggregate reactions, especially in AACs with reactive aggregates [44]. Likewise, limestone is a relatively low-cost and widely available material, which makes it economically viable as a partial substitute in AACs. Its incorporation can reduce the reliance on high-cost alkaline activators, making the production of AACs more cost-effective and sustainable, which are critical factors for achieving scalable industrial applications [37].

The CaO content of the raw material strengthens the alkali-activated matrix by forming an amorphous-structured Ca-Al-Si gel type. Also, calcium has a positive effect on the compressive strength in alkali-activated binders because when the CaO content is high, the structural porosity decreases, and the resulting formation of the amorphous-structured Ca-Al-Si gel strengthens the final reaction product [45,46,47,48]. Calcium compound additions, such as limestone (CaCO_3_), in metakaolin activated by NaOH result in the formation of alkaline aluminosilicate binders, which exhibit good strength in their hardened state. Also, it has been found that adding limestone to alkali-activated slag/ash binary systems improves workability in the fresh state. The use of limestone leads to a shorter setting time. An attractive advantage is that limestone dissolution can be increased using low concentrations of alkaline activators. The addition of CaCO_3_ in aluminosilicate mixtures can lead to the formation of monocarboaluminate phases in alkali-activated systems since it has been found that there is a dissolution of Ca^2+^ from CaCO_3_ in limestone [49,50,51,52,53].

Based on the above, this work aimed to study the effect of μ-limestone additions in slag and binary alkali-activated cement (slag–fly ash) and investigate the leaching of Ca^2+^ ions from μ-limestone in a Na(OH) alkaline solution. Also, it studies how Ca^2+^ ions influence the reaction kinetics of hybrid hydration gels and their influence on mechanical characteristics.

## 2. Materials and Methods

### 2.1. Material Characterization

**Figure 1:**
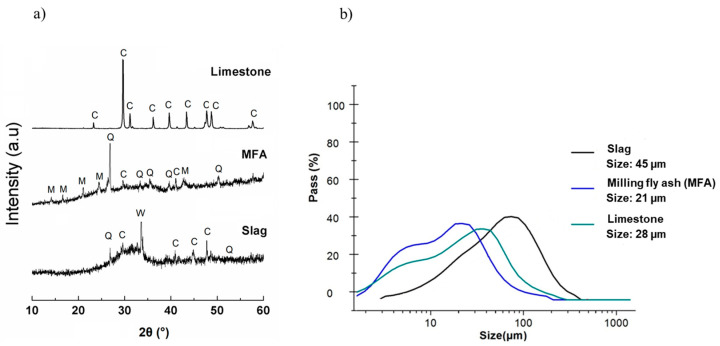
(**a**) Diffractograms of raw materials (limestone—LSP, ground fly ash—MFA, and slag—S). C, M, Q, and W refer to limestone, mullite, quartz, and merwinite, respectively. (**b**) Particle size distribution of raw materials (limestone—LSP, ground fly ash—MFA, and slag—S).

The precursors utilized to produce geopolymers included (i) granulated blast-furnace slag sourced from the Lazaro Cardenas steel plant in Michoacán, Mexico, (ii) Class F fly ash (FA) obtained from the Nava thermoelectric facility in Coahuila, Mexico, and (iii) micro-limestone extracted from mountain deposits in Nuevo León, Mexico. An alkaline solution was prepared using sodium hydroxide from Jalmek Cientifica, S.A. de C.V., with a purity of 98.5% (San Nicolás de los Garza, Nuevo León, Mexico) and bi-distilled water. Before employing the GGBFS, FA, and limestone as precursor materials in the alkali-activated cement, a 1 h grinding process was conducted in a vibro-energy ball mill (DM 1 model, Sweco Inc., Stockholm, Sweden), utilizing 80 kg of 20 mm steel balls along with 5 kg of the raw material in the mill. After the grinding process, the raw material was analyzed via X-ray fluorescence (XRF) using a Panalytical X-ray fluorescence spectrometer (XRF), Epsilon 3XL, Almelo, The Netherlands (Table 1). Meanwhile, the structural properties were determined via X-ray diffractometry (XRD) using an equipment Bruker D8 Advance model (Karlsruhe, Germany) with a Vantec detector and Cu Kα radiation (λ = 1.5406 Å), operated at 40 kV, 40 mA, and a scanning rate of 0.05 (2θ/s) at an interval of 2θ from 10° to 60° (Figure 1a).

In Figure 1a, C, M, Q, and W refer to limestone (identified as C, JCPDS 08-30577, JCPDS 08-62335), mullite (identified as M, JCPDS 08-41205), quartz (identified as Q, JCPDS 07-81253), and merwinite (identified as W, JCPDS 03-50591), respectively. The average particle sizes (d_50_) of the slag (S), ground fly ash (MFA), and µ-limestone (LSP) were 45 μm, 21 μm, and 28 μm, respectively, as identified by the laser diffraction granulometry method (HORIBA laser model LA-950, Irvine, CA, USA) (see Figure 1b for the particle size distribution). The raw materials were homogenized for 1 h using a two-dimensional rotary drum powder mixer (Higao Tech, Shanghai, China).

### 2.2. Mix Design

Table 2 shows the proportions and dosages of the mixtures used. Mixtures of 500 g (from each sample) were mixed in a rotating homogenate drum to obtain a uniform distribution of particles and, thereby, better alkaline activation. The pastes with the highest slag content were activated with NaOH-4M and cured at 25 °C under controlled humidity (80% RH) for 28 days.

The activator solution was prepared with reagent-grade sodium hydroxide (4M) (Jalmek Cientifica, S.A. de C.V.). The workability of the pastes was measured according to ASTM C 230-14 (UNE EN 1015-3). The mixtures were poured into 10 × 10 × 60 mm^3^ metal molds. The molds (in fresh state) were placed on a vibratory table for 15 s to obtain better packing and avoid high porosity.

### 2.3. Instrumental Methods

The compressive strength was evaluated at 1, 3, 7, and 28 days of age on cubic specimens with a side length of 100 mm in an IBERTEST Autotest 250/10 testing machine, Madrid, Spain. The values presented were determined as the average of six samples for each paste system. The compressive strength was measured according to ASTM C 109-07. The machine was programmed to apply a compressive force at 20 kN/s (2 MPa/s) for these specimens.

The flexural strength was evaluated at 1, 3, 7, and 28 days of age on 10 × 10 × 60 mm prism specimens in a DMA GABO Eplexor NETZSCH bending machine (Selb, Germany). The final flexural strength value was reported as the average of six specimens for each paste system. The flexural strength was measured according to ASTM C 293-16. The load rate was 15 N/min (σ = 0.9 MPa).

After the mechanical tests at their respective ages, samples were collected to be analyzed by Fourier-transform infrared spectroscopy (FTIR) using an ATTI MATTSON Genesis and a Nicolet 6700 from Thermo Scientific, Waltham, MA, USA.

The microstructure was analyzed using the scanning electron microscopy technique (SEM) utilizing an SEM (model JSM 6490LV, JEOL Ltd., Tokyo, Japan) microscope with an acceleration voltage of 20 kV. The SEM was equipped with an Energy-Dispersive X-ray Spectrometer (EDS) (INCA-Sight from OXFORD instruments, Abingdon, Oxfordshire, UK) for semiquantitative and semiqualitative microanalyses.

The heat flow and hydration reactions were analyzed using isothermal calorimetry with a TAM Air calorimeter from TA Instruments, New Castle, DE, USA. Isothermal calorimetry experiments were carried out with an ASTM C 1679. The pastes were mixed externally and loaded into the isothermal calorimeter. The time elapsed between the instant the activating solution was added to the powder and the paste being loaded into the calorimeter was around 2–3 min. The tests were run for 100 h with the calorimeter set at 25 °C.

A complementary experiment was conducted to determine Ca^2+^ leaching in alkaline aqueous solutions. For this purpose, limestone powders were separately added to water and NaOH-4M solutions. Subsequently, the powders were washed and analyzed using FTIR, XRD, and SEM.

## 3. Results

### 3.1. Limestone Study in Aqueous Solutions

**Figure 2:**
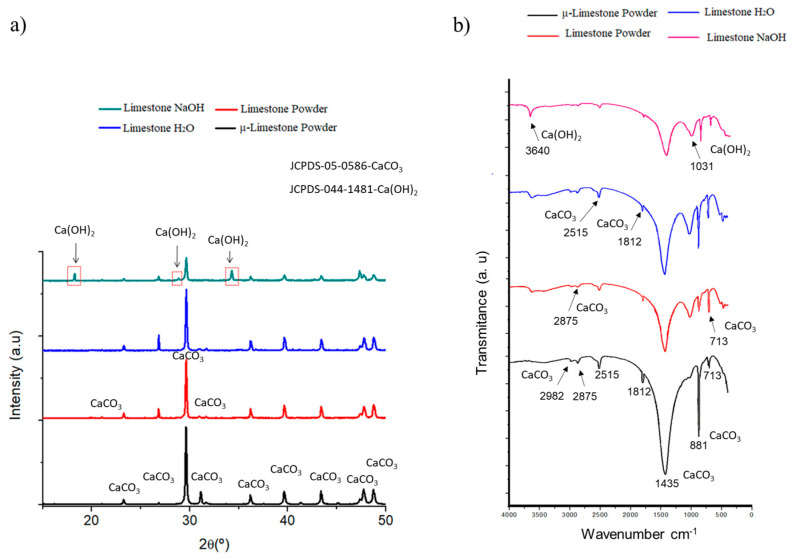
(**a**) X-ray diffraction and (**b**) FTIR analyses of treatment of limestone powders in different solutions. CaCO_3_ (713, 881, 1435, 1812, 2515, 2875, and 2982 cm^−1^). Ca(OH)_2_ (1031 and 3640 cm^−1^).

First, it was confirmed that the μ-limestone remains inert in neutral solutions. In contrast, when exposed to alkaline solutions, the μ-limestone undergoes a dissolution reaction. The μ-limestone was analyzed using X-ray diffraction (XRD). Figure 2a presents the peaks coinciding with the portlandite phase (JCPDS -044-1481). The portlandite peaks are present at 2θ angles 16°, 29°, and 34°. The black and red lines show the limestone in a dry environment. The blue line shows the limestone immersed in water, and the green line shows the limestone embedded in NaOH at 4M.

An FTIR analysis proved the limestone’s reactivity in a highly alkaline system. Figure 2b shows the characteristic bands of calcite-CaCO_3_ at 713 cm^−1^, 881 cm^−1^, 1435 cm^−1^, 1812 cm^−1^, 2515 cm^−1^, 2875 cm^−1^, and 2982 cm^−1^. Portlandite was found in the 1031 cm^−1^ and 3640 cm^−1^ bands. The band’s intensity at 3640 cm^−1^, corresponding to Ca(OH)_2_, increases upon exposure to NaOH, since this band has lower intensity with µ-limestone powder and limestone powder, and also in limestone exposed to H_2_O. Although the appearance of portlandite in the 3640 cm^−1^ band of the dry limestone powders and those exposed to water and alkaline solution may be due to the moisture adsorbed in the powders, the change of intensities indicates more reaction with the alkaline solution, giving a higher intensity to the portlandite phase. In addition, it is observed that in the FTIR analysis corresponding to the limestone exposed to NaOH, the limestone bands decrease in contact with the alkaline solution as the Ca(OH)_2_ bands increase. Therefore, the interaction of the alkaline solution with the limestone could be taking place, transforming it into portlandite–Ca(OH)_2_. This spectroscopy analysis proves that limestone is not inert in highly alkaline systems. There is a high degree of reactions of CaCO_3_ limestone into Ca(OH)_2_ in low-molarity solutions.

**Figure 3:**
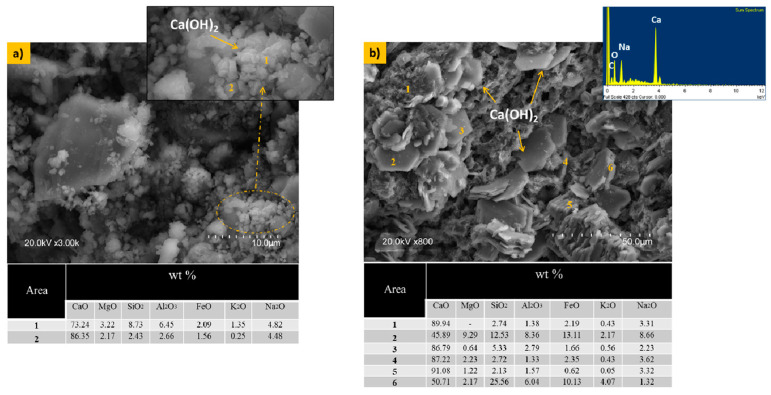
The microstructure of (**a**) μ-limestone submerged in water and (**b**) μ-limestone submerged in NaOH for 28 days and the variation in chemical composition in terms of the mineralogical nature of the limestone investigated by EDS microanalysis.

Figure 3 shows a microstructural analysis of the limestone particles immersed in water and sodium hydroxide (NaOH) alkaline solution for 28 days. Figure 3a shows the limestone immersed in water, where a slight reaction of hexagonal particle formation corresponding to the portlandite phase can be observed. Meanwhile, Figure 3b shows the limestone immersed in an alkaline solution of sodium hydroxide (NaOH) for 28 days, where the formation of portlandite (hexagonal particles) is more pronounced. Therefore, it can be concluded that the reactivity of limestone when immersed in an alkaline solution (NaOH) leads to the formation of portlandite. Also, the variation in chemical composition in terms of the mineralogical nature of the limestone was investigated by microanalysis.

According to that discussed above, it is confirmed that the reaction product between calcium carbonate (CaCO_3_) and sodium hydroxide (NaOH) is mainly the formation of calcium hydroxide Ca(OH)_2_ in the form of portlandite. Ca(OH)_2_ plays a significant role in influencing the properties of alkali-activated cements. As a source of calcium ions, Ca(OH)_2_ enhances the formation of calcium–silicate–hydrate (C-S-H) and calcium–aluminosilicate–hydrate (C-A-S-H) gels, which are essential for early strength and durability in AAC systems [38,39,42,54]. Other authors recognize the formation of sodium carbonate (Na_2_CO_3_).

CaCO_3_(s) + 2NaOH(aq)→Ca(OH)_2_(aq) + Na_2_CO_3_(aq)

Pingitore Jr. et al. studied CaCO_3_ particles of two size ranges—62–125 μm (fine) and 250–500 μm (coarse)—in their aragonite and calcite forms within an alkaline solution. Their findings showed that adding CaCO_3_ powders, regardless of the crystalline phase, resulted in the dissolution of carbonate into calcium hydroxide. Additionally, smaller particle sizes exhibited higher reactivity in the alkaline solution, emphasizing the impact of particle size on reactivity [55].

Limestone (CaCO_3_) is frequently added to AACs to enhance reactivity, given that it readily dissolves under alkaline conditions to supply Ca^2+^ ions. CaCO_3_ raises the availability of calcium ions, which, under alkaline conditions, contribute significantly to the formation of C-S-H gels. This C-S-H formation improves binding within the matrix, enhancing mechanical properties. The addition of CaCO_3_ promotes the densification of the binder phase by reacting with silicate and aluminate precursors to form a more interconnected C-S-H and C-A-S-H network. This results in a more refined microstructure with reduced porosity, which improves durability, especially in aggressive environmental conditions [56].

CaCO_3_ can partially react, producing calcium–silicate–hydrate (C-S-H) phases along with the typical aluminosilicate network in alkali-activated cements. This C-S-H gel provides additional binding and can improve compressive strength. When combined with other pozzolanic materials, Ca^2+^ ions from limestone may promote additional gel phases, including C-A-S-H (calcium–aluminosilicate–hydrate), which strengthens the alkali-activated cement. As has been mentioned, calcium from limestone can react with aluminosilicate components to form secondary phases like C-S-H or C-A-S-H gels. These gels promote benefits in geopolymers with high calcium content since they supplement the binding phases and help create a hybrid gel structure. This dual gel network enhances strength, improves durability, and presents better resistance to carbonation and chemical attacks, as it lowers the matrix’s permeability [41].

Finally, adding limestone as a partial substitute to alkali-activated cements or geopolymers can influence production costs in several ways. Limestone is generally more abundant and less expensive (USD 50–100 per ton) compared to other raw materials like metakaolin (USD 300–650 per ton) or fly ash (USD 500–850 per ton), which can lead to reduced material costs [57,58,59,60,61,62]. Additionally, incorporating limestone can sometimes improve workability and reduce the need for high-temperature curing, which may lower energy costs. However, balancing limestone content is crucial, as excessive amounts could negatively impact the material’s durability and mechanical properties, potentially requiring additional modifications that could offset initial savings.

### 3.2. Mechanical Testing

#### 3.2.1. Compressive Strength

**Figure 4:**
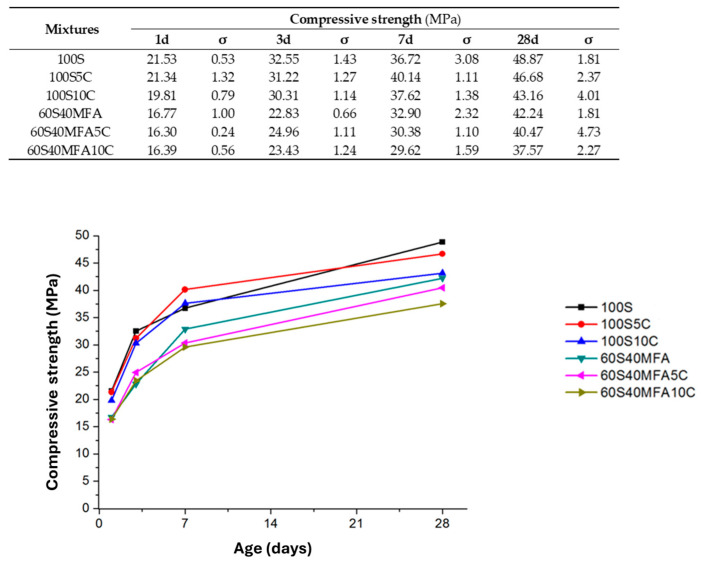
Compressive strength results.

The results of the compression tests for the experimental pastes activated with NaOH-4M are presented in Figure 4. As expected, the highest strength was achieved by the pure slag pastes. Studies have shown that alkali-activated ground granulated blast-furnace slag (GGBFS) provides several advantages, including rapid strength development, improved mechanical properties, excellent durability, and superior resistance to chemical attack [63]. The activation of slag by alkalis depends on the ability of the alkalis to effectively solubilize silica and alumina from the slag [23,46]. Higher alkalinity, characterized by increased concentrations of OH^−^ ions in a solution, enhances the dissociation of silica (Si) and the release of calcium (Ca). This process increases the potential for forming greater amounts of strength-contributing reaction products, such as C-S-H and C-A-S-H-type gels. These gels improve the setting properties and mechanical strength of alkali-activated cement by reducing porosity and promoting densification within the cementitious matrix [64,65,66,67,68].

As observed, adding limestone to the slag pastes did not improve the mechanical properties at 1 d and 3 d, since the pastes with 5% and 10% limestone addition at 1 d decreased by approximately 0.88% (21.34 MPa) and 7.98% (19.81 MPa), respectively, compared to the 100S pastes (21.53 MPa). Meanwhile, the pastes with 5% and 10% limestone addition at 3 d decreased by approximately 4.08% (31.22 MPa) and 6.88% (30.31 MPa), respectively, compared to the 100S pastes (32.55 MPa). However, the analysis shows that at 7 d, the 100S5C paste increased by 9.31% (40.14 MPa) compared to the 100S paste (36.72 MPa), while the 100S10C paste at 7 d increased by 2.45% (37.62 MPa) compared to the 100S paste. At 28 d, the 100S5C paste decreased by 4.48% (46.68 MPa) compared to the 100S paste (48.87 MPa). Meanwhile, the 100S10C paste at 28 d decreased by 11.68% (31.16 MPa) compared to the 100S paste.

In contrast, fly-ash-based alkali-activated concrete has attracted considerable interest due to its widespread availability, high alumina and silica content, and low water demand [69,70,71,72,73]. However, these concretes need curing at elevated temperatures of around 60 °C for effective geopolymerization, as fly ash exhibits low reactivity under ambient conditions [74]. The exclusive use of fly ash for producing alkali-activated concrete is limited in structural applications due to its requirement for ambient curing. As is known, to address this limitation, ground granulated blast-furnace slag is added to fly-ash-based alkali-activated cement. The addition of slag eliminates the need for high-temperature curing, as its calcium oxide (CaO) content enhances strength by promoting the transformation from a N-A-S-H gel structure to a (N, C)-A-S-H structure under normal conditions. Moreover, slag inclusion has been demonstrated to significantly improve the compressive strength of fly-ash-based alkali-activated concrete [13,21].

As shown in Figure 4, the compressive strength of the pastes decreases with increasing fly ash content. This reduction is attributed to the low reactivity of fly ash at ambient temperatures, primarily due to its lower content of reactive glassy phases compared to slag [75,76,77]. To achieve acceptable compressive strength, higher curing temperatures or more highly alkaline solutions are necessary to dissolve the glassy phases in fly ash and promote the formation of strength-contributing reaction products. In addition, as the content of fly ash increases, the specific surface area of the fly ash becomes too large, the liquid-phase environment of the alkali-activated reaction is insufficient, the reaction of the fly ash particles is inadequate, and the generated gel product decreases accordingly. As a result, the overall microstructure is relatively loose, and the strength of the material will also decrease [78]. As observed, the 60S40MFA pastes (without limestone addition) developed more strength than the pastes with limestone addition, except for the strength developed at 3 d, where the 60S40MFA5C and 60S40MFA10C pastes improved in strength by 9% (24.96 MPa) and 2% (23.43 MPa), respectively, when compared to the 60S40MFA paste (22.83 MPa). Pastes with 5% limestone additions showed higher mechanical behavior than those with 10% additions.

Despite the strength not being improved by adding limestone to the 100S and 60S40MFA pastes, all compressive strengths developed for all pastes were higher than 20 MPa, which is the lowest compressive resistance demand of an OPC 20R (ASTM C 150-95 [79]; Standard Specification for Portland Cement). Mechanically, these pastes find applications in mortars, industrial floors, masonry elements, soil stabilization, etc. In addition, these pastes might find applications as the cementitious matrixes of new basal fiber-reinforced cement composites [80].

#### 3.2.2. Flexural Strength

**Figure 5:**
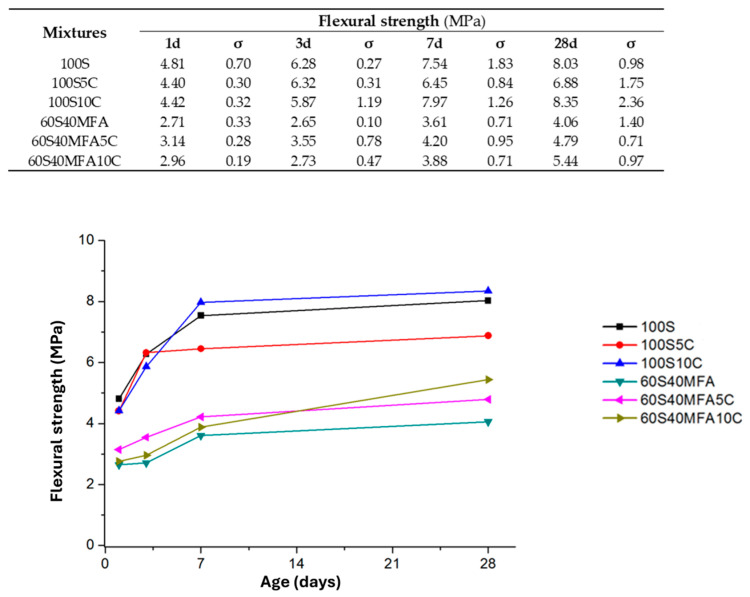
Flexural strength results.

The results of the flexural tests for the pastes activated with NaOH-4M are shown in Figure 5. As shown, pastes with 100% slag content developed higher strength than the slag pastes with limestone additions (5% and 10%), except for the strength developed at 28 d, where the 100S10C pastes slightly improved in strength by 3.98% (8.35 MPa) when compared to the 100S paste (8.03 MPa). Meanwhile, the paste with the highest flexural strength at 1 d (3.14 MPa), 3 d (3.55 MPa), and 7 d (4.2 MPa) was the 60S40MFA5C paste. On day 28, the 10% limestone addition to the 60S40MFA paste (4.06 MPa) improved the flexural strength by about 13.39% (5.44 MPa), resulting in the highest flexural strength.

There was an improvement in flexural strength as the limestone was added to the pastes. According to Idawati et al. [81], this behavior can be attributed to the gel formation of a slag-based geopolymer governed by a C-A-S-H-type gel, which improves the setting and strength characteristics of alkaline-activated cement via porosity reduction and the densification process of the cementitious matrix. Meanwhile, fly-ash-based alkali-activated cement was controlled by a N-A-S-H-type gel. It has been found that C-A-S-H- and N-A-S-H-type gels can coexist when slag/fly ash composites are mixed. A hybrid-type gel (N, C)-A-S-H is also identified in slag systems due to the calcium released by the dissolution of the slag and its incorporation into the N-A-S-H-type gel because of fly ash activation [10,82]. According to the results of our study, it is postulated that a (N, C)-A-S-H-type gel is formed as a part of the Ca^2+^ released by the dissolution of the limestone and its incorporation into the N-A-S-H gel due to fly ash activation. It has been reported that the presence of (N, C)-A-S-H gel improves the strength, durability, and carbonation resistance in AACs and geopolymers [37,38,41]. Another aspect to consider is that C-S-H gel is more rigid than N-A-S-H and (N, C)-A-S-H gels. Therefore, a cement matrix containing N-A-S-H and (N, C)-A-S-H gels could have more flexibility than a cement matrix composed of C-S-H when subject to flexural stresses.

### 3.3. Microstructural Analysis

**Figure 6:**
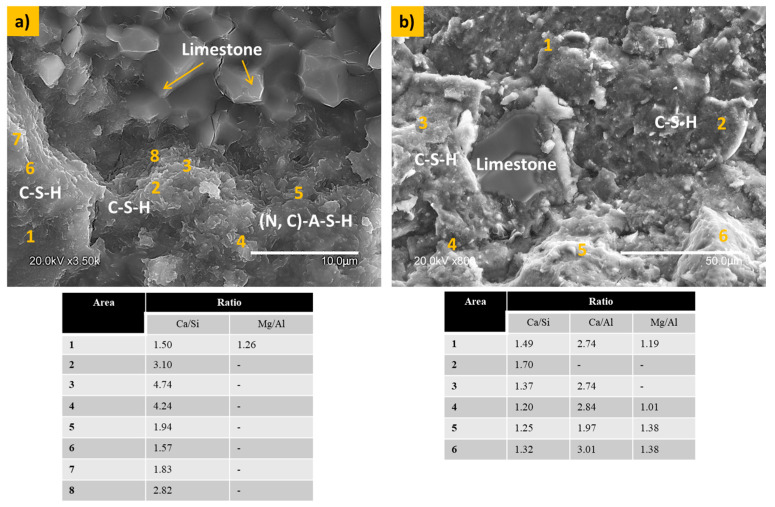
Microstructure of the 100S10C paste. (**a**) Distribution of the limestone embedded in the cementitious matrix and (**b**) interaction of limestone with the slag paste.

The effect of μ-limestone addition to the alkali-activated cement (AAC) (the interaction between slag particles and ground fly ash) on the hydration process of the paste with the highest limestone content was analyzed by SEM. Figure 6 illustrates the microstructure of the 100S10C paste. Figure 6a depicts the dispersion of the limestone integrated within the cementitious matrix. The 100S10C paste presented a homogeneous and close-grained structure, where a Ca/Si ratio (calculated according to the microanalysis) was found between 1.5 and 4.7. These ratios favor the C-S-H gels, which results in a very structurally dense matrix. In terms of the Ca/Si ratio (by microanalysis), an ideal Ca/Si ratio in some zones for (N, C)-A-S-H gel formation was observed (a Ca/Si ratio around 1.0–1.3). (N, C)-A-S-H gel is a complex calcium–aluminosilicate–hydrate gel that incorporates sodium and carbonate ions within its structure. Also, the microanalysis of some specific zones provides a Mg/Al ratio with values between 1 and 1.2, corresponding to hydrotalcite phase formation.

Figure 6b shows the microstructural interaction of limestone with the slag paste. Some unreacted limestone particles are embedded in a matrix composed of hydration gels. Gel formation was established based on the Ca/Si ratios. The chemical interaction is assessed based on stoichiometric reactions, specifically the Ca/Si ratios. The Ca/Si ratio between 1.2 and 1.7 confirmed the presence of C-S-H gels. Low Ca/Si ratios promote the (N, C)-A-S-H gel in some zones. A Mg/Al ratio between 1 and 1.38 suggests hydrotalcite phase formation.

**Figure 7:**
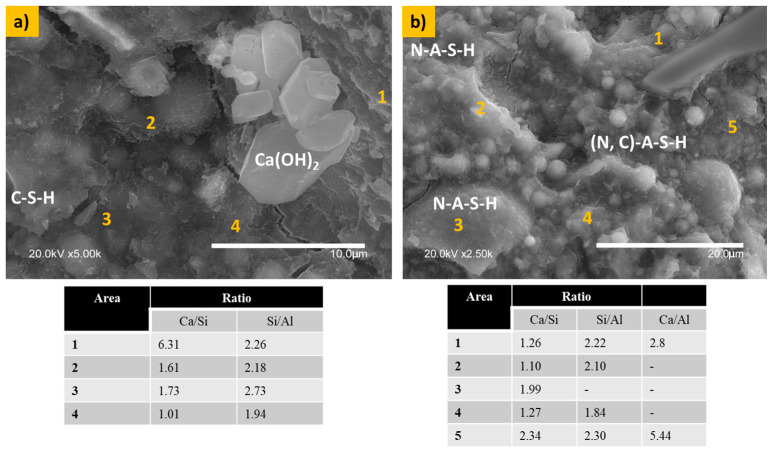
Microstructure of the 60S40MFA10C paste. (**a**) shows the interaction of limestone with hybrid hydration gels. (**b**) shows the dissolution of the particles and the formation of hydration gels such as the N-A-S-H gel and hybrid (N, C)-A-S-H gel formation.

Figure 7 illustrates the microstructure of the 60S40MFAS10C paste. The presence of μ-limestone significantly influences the microstructural properties by promoting the formation of hydration gels. As discussed before, µ-limestone dissolves in alkaline solutions, which results in the leaching of Ca(OH)_2_ particles. Ca(OH)_2_ reacts with the N-A-S-H gels from the activated fly ash, forming (N) C-A-S-H gels. One of the dominant reaction products in the 60S40MFAS10C paste is the N-A-S-H gel. Figure 7a shows Ca(OH)_2_ particles with a well-defined morphology (hexagon-like morphology); the precipitation of these particles originated from the reaction of limestone with sodium hydroxide. Ca/Si ratios between 1 and 6.3 favor C-S-H gel formation. Figure 7b shows the presence of N-A-S-H and hybrid (N, C)-A-S-H gels. Lower Ca/Si ratios (0.5–1.0) and Si/Al ratios ≥ 2 lean towards sodium–aluminosilicate–hydrate (N-A-S-H) gel formation. Meanwhile, a Ca/Si ratio of 1.0–1.3 is optimal for (N, C)-A-S-H gels.

**Figure 8:**
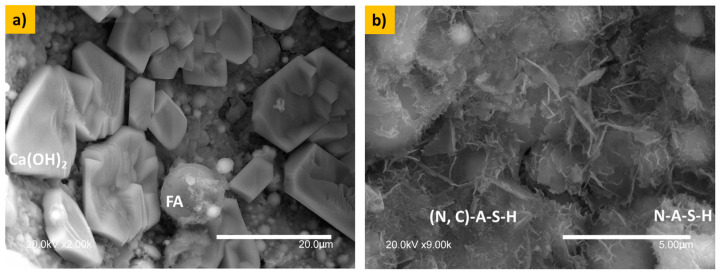
Microstructure of the 60S40MFA10C paste. (**a**) shows the formation of a well-defined portlandite-Ca(OH)_2_ with hexagon-like morphology. (**b**) shows the formation of (N, C)-A-S-H and N-A-S-H gels.

Figure 8a shows the formation of a well-defined portlandite (Ca(OH)_2_) with hexagon-like morphology interacting with fly ash particles. Figure 8b shows the formation of (N, C)-A-S-H and N-A-S-H gels thanks to the high sodium concentrations. (N, C)-A-S-H gel formation and N-A-S-H gel formation were hypothesized according to the Si/Al and Ca/Si ratios.

In alkali-activated cements and geopolymers, the gel formation is primarily influenced by the stoichiometric ratios of silicon (Si), aluminum (Al), calcium (Ca), magnesium (Mg), and sodium (Na) in the source materials. These ratios are essential in determining the type and properties of the gels formed, influencing strength, durability, and setting characteristics.

The Si/Al ratio is crucial in alkali-activated reactions and geopolymerization. A higher Si/Al ratio (>4) generally leads to more silicate bindings, forming a three-dimensional network structure. Low Si/Al ratios (1–2) result in more brittle and rigid structures, while moderate-to-high ratios (2–4) enhance flexibility and strength. A balanced Si/Al ratio around 2–3 is typically sought for optimal mechanical properties in alkali-activated cements and geopolymers [83].

The Al/Si ratio affects the cross-linking density within the gel network. Higher Al/Si ratios can increase the degree of cross-linking, which can improve thermal resistance and durability but may reduce workability. Ratios are adjusted depending on the application requirements, e.g., gels that are more cross-linked (higher Al/Si) or less rigid (lower Al/Si) [84].

In systems with calcium, such as alkali-activated slags, the Ca/Si ratio influences the formation of calcium–silicate–hydrate (C-S-H) gels, which contribute to early strength development. A higher Ca/Si ratio promotes C-S-H gel formation rather than sodium aluminosilicate hydrate (N-A-S-H) gels, which are typical in geopolymer systems. Lower Ca/Si ratios favor N-A-S-H gel formation, which is more characteristic of traditional geopolymer and alkali-activated systems and imparts different durability and chemical resistance properties [38].

The Ca/Si ratio for C-S-H formation in alkali-activated cements ranges from 0.5 to 2.0. Although, the most common Ca/Si ratio range for C-S-H formation is 0.8–1.5. In AACs, a higher Ca/Si ratio favors the formation of calcium–silicate–hydrate (C-S-H) gel, which is crucial for early strength. Lower Ca/Si ratios (0.5–1.0) lean towards sodium–aluminosilicate–hydrate (N-A-S-H) gel formation, which is more typical in traditional geopolymer and alkali-activated matrices, promoting long-term durability and chemical resistance; however, these gels generally result in slower early strength gain [37].

The Mg/Al typical range ratio for alkali-activated cements and geopolymers often falls within the range of 0.1–1.0. This range supports the formation of magnesium-containing gels, like magnesium–aluminosilicate–hydrate (M-A-S-H) phases. M-A-S-H gels enhance the matrix’s resistance to shrinkage and provide additional durability in high-temperature applications. Lower Mg/Al ratios (around 0.1–0.3) are associated with gels that are more structurally stable and compatible with other phases, such as C-A-S-H and N-A-S-H. Ratios closer to 1.0 increase magnesium participation, potentially forming layered double hydroxides that improve chemical stability but may reduce early strength [85].

The Na/Si typical range ratio for alkali-activated cements and geopolymers is often estimated within the range of 0.4 to 1.5. Lower Na/Si ratios (0.4–0.6) favor denser N-A-S-H gels with high cross-linking, contributing to better durability and chemical resistance. Higher Na/Si ratios (1.0–1.5) can enhance the dissolution of aluminosilicate precursors, increasing the availability of silicate species, but may also increase the porosity of the final matrix. Lower Na/Si ratios result in slower reaction kinetics. Meanwhile, higher Na/Si ratios accelerate the reaction rate and setting time, which can improve early strength but may compromise long-term stability [56].

In alkali-activated cements and geopolymers, the (N, C)-A-S-H phase refers to a complex calcium–aluminosilicate–hydrate gel that incorporates both sodium (N) and carbonate (C) ions within its structure. This type of gel formation is notable in systems that include calcium-rich precursors, such as ground granulated blast-furnace slag (GGBFS), and is activated in highly alkaline environments (pH > 12) through the addition of sodium hydroxide or sodium silicate as an activator in AAC systems. The presence of sodium and carbonate ions modifies the gel’s properties, impacting strength, durability, and carbonation resistance in AACs and geopolymers. A Ca/Si ratio around 1.0–1.3 is generally ideal for (N, C)-A-S-H gels [37,38,41].

### 3.4. X-Ray Diffraction

**Figure 9:**
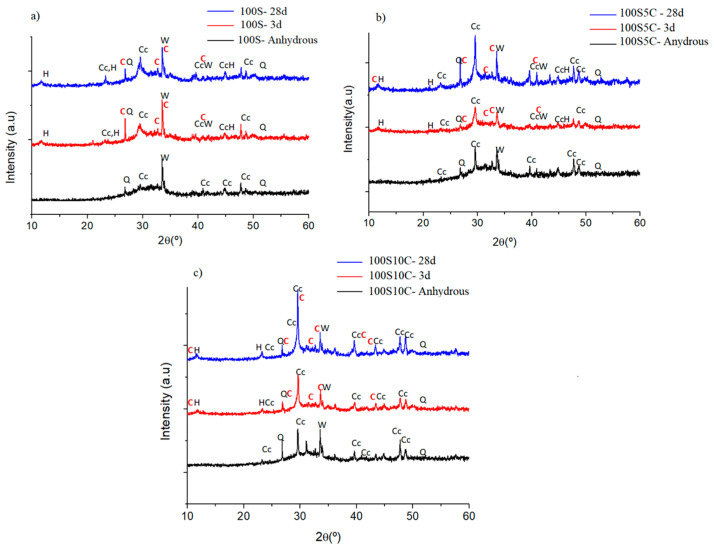
Mineralogical formation of compounds in the 100S paste with and without the addition of limestone. (**a**) 100S paste, (**b**) 100S5C paste, and (**c**) 100S10C paste. Calcite (Cc), quartz (q), mullite (M), merwinite (W), tobermorite (C), and hydrotalcite (H).

Experimental paste diffractograms are plotted in Figure 9. Figure 9a shows the 100S paste, Figure 9b shows the diffractograms of the 100S5C paste, and Figure 9c shows the 100S10C paste. All pastes were activated with NaOH-4M at an L/S ratio of 0.30 and cured at 25 °C.

The analysis shows the diffractograms corresponding to the anhydrous mixtures (black color) in all conditions, where the crystalline phases identified correspond to the characteristic crystalline phases of the slag: calcite (identified as Cc, JCPDS 08-30577, JCPDS 08-62335), quartz (identified as Q, JCPDS 07-81253), mullite (identified as M, JCPDS 08-41205), and merwinite (identified as W, JCPDS 03-50591). In the 100S pastes hydrated for 3 days (red color) and 28 days (blue color), new crystalline compounds resulting from the hydration reactions were observed: tobermorite (identified as C, JCPDS 01-0896458, JCPDS 02-90373) and hydrotalcite (identified as H, JCPDS 41-1428). For the 100S10C pastes with the higher limestone addition, the highest intensity signals were due to calcite (identified as Cc, JCPDS 08-62335), and at the 2θ angle 31°, the C-S-H gel phase known as tobermorite was identified (identified as C, JCPDS 02-90373).

**Figure 10:**
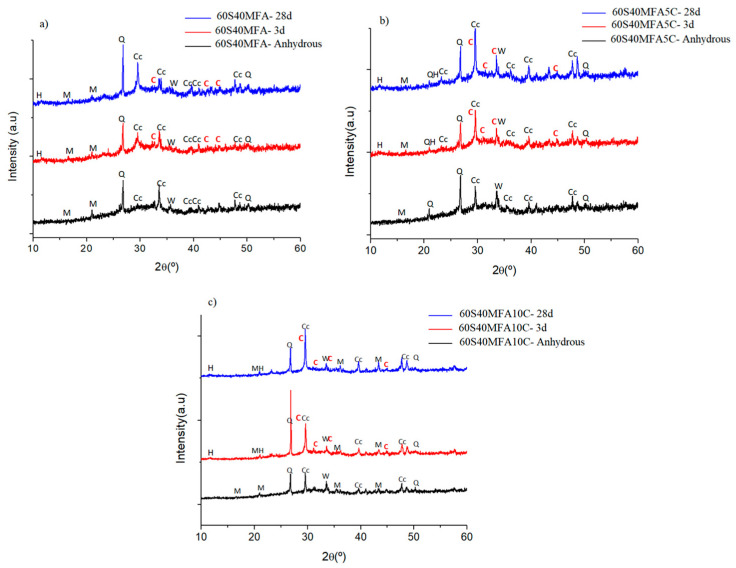
Mineralogical formation of compounds in the 60S40MFA paste with and without the addition of limestone. (**a**) 60S40MFA paste, (**b**) 60S40MFA5C paste, and (**c**) 60S40MFA10C paste. Calcite (Cc), quartz (q), mullite (M), merwinite (W), tobermorite (C), and hydrotalcite (H).

Figure 10a–c show the diffractograms of the 60S40MFA, 60S40MFA5C, and 60S40MFA10C pastes, respectively, for the following three conditions: without hydration, 3 d of curing, and 28 d of curing. As observed, the addition of limestone does not lead to the formation of new crystalline phases. The analysis shows that the 60S40MFA5C and 60S40MFA10C pastes present the same crystalline phases, i.e., the same reaction product from the hydration process at 3 and 28 days of curing (calcite, quartz, mullite, merwinite, tobermorite, and hydrotalcite).

### 3.5. FTIR Analysis

The Fourier-transform infrared spectroscopy (FTIR) study of the anhydrous slag shows a band identified between 950 and 1100 cm^−1^ associated with Si-O-T asymmetric stress vibrations. T can be either Si or Al from the Si-O bonds of the silicon tetrahedra, whose bandwidth indicates a higher structural disorder for this paste. This band was identified at 980 cm^−1^. Meanwhile, the band between 510 and 515 cm^−1^ is attributable to Si-O-Si bending vibrations (SiO_4_^2−^ tetrahedra). Between 600 and 800 cm^−1^, a band is identified due to the vibrations of the Al-O bonds of the AlO_4_ group in the slag. At 600 cm^−1^, bands are identified due to Si-O-Si bending vibrations. The bands from 1140 to 1480 cm^−1^ are associated with the asymmetric strain of (CO_3_)^2−^ carbonates. At 875 cm^−1^, a small “shoulder” appears due to the out-of-plane bending of (CO_3_)^2−^. The OH water stress and strain bands are identified at 3400 cm^−1^ and 1650 cm^−1^, respectively [86].

**Figure 11:**
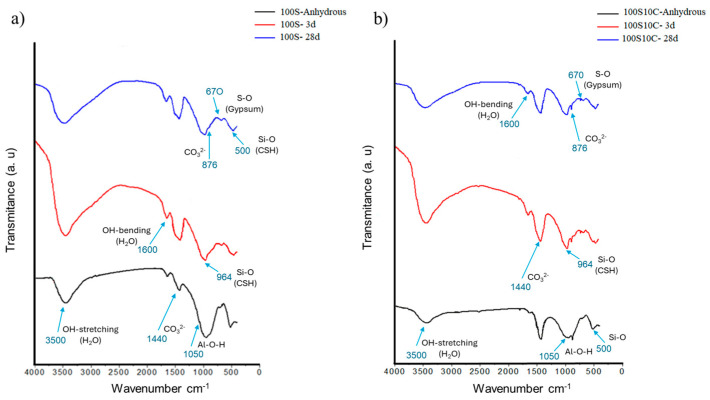
(**a**) FTIR spectra of 100S paste and (**b**) 100S510C paste.

Figure 11a,b show the FTIR spectra corresponding to the blast-furnace slag pastes (100S) and blast-furnace slag with 10% limestone addition (100S10C), respectively. For the 100S paste, a band identified at 1440 cm^−1^ is attributed to the (CO_3_)^2−^ band, indicating the presence of (CO_3_)^2−^ carbonates. This band is detected both in the anhydrous slag and in the alkaline-activated slags, with higher intensity in the pastes with limestone addition in the range from 1440 to 1480 cm^−1^. In the FTIR spectra of slag pastes activated with NaOH-4M, the bands at 876 and 712 cm^−1^ are associated with vibrations of carbonates in the form of calcite. In the 700–650 cm^−1^ region, the bands associated with the vibrations of Al-O bonds in silicoaluminate glasses are detected. Figure 11b shows a band at 876 cm^−1^ corresponding to unreacted slag. The absorption at 670 cm^−1^ corresponds to the vibrations of gypsum at 3 days and 28 days of hydration. An associated band characteristic of the C-S-H gel can be observed at 500 and 964 cm^−1^. A pronounced band at 1440 cm^−1^ is attributed to the vibrational mode of the O-C-O bonds in the (CO_3_)^2−^ group, resulting from the addition of limestone. Finally, at 3500 cm^−1^ and 1600 cm^−1^, the tension (O-H) and deformation (H-OH) bands of water appear, respectively. These bands do not appear in the spectrum of the anhydrous slag. In the spectra of the activated slag pastes, these bands indicate the presence of water, crystallization, or adsorption molecules in the reaction products formed. All significant variations mainly occur in the presence of calcium carbonate. However, the FTIR spectra of the different samples studied are very similar. In all cases, a broad band between 950 and 1000 cm^−1^ is observed, corresponding to the asymmetric stretching vibrations of Si-O bonds within the SiO_4_ tetrahedra. At 1050 cm^−1^, a band is associated with the vibrational mode of Al-O-H. At 450–500 cm^−1^, another broad band attributable to the deformation vibrations (O-Si-O) of the same group of SiO_4_ tetrahedra is observed [87,88].

**Figure 12:**
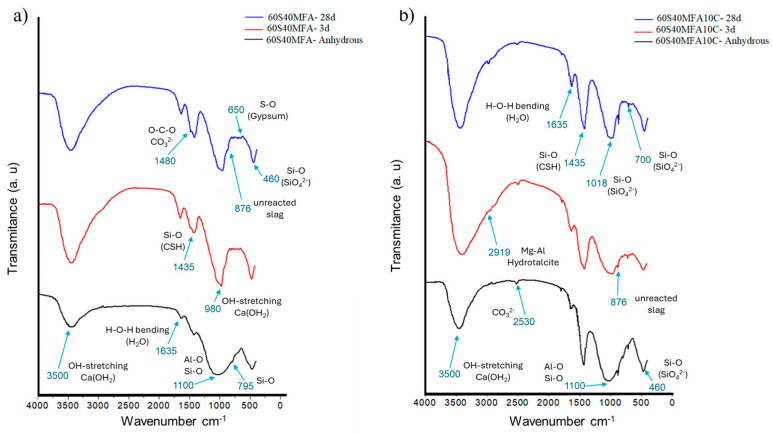
(**a**) FTIR spectra of 60S40MFA paste and (**b**) 60S40MFA10C paste.

Figure 12 shows the FTIR spectra of the 60S40MFA (a) and 60SMFA10C (b) pastes. Figure 12a shows a band at 460 cm^−1^ corresponding to the Si-O characteristic of unreacted ash, which is observed in the anhydrous mixture and the hydrated pastes at 3 and 28 days. Meanwhile, between 1100 cm^−1^ and 795 cm^−1^, a band associated with the asymmetric tension of the Si-O-T bonds (T: Si or Al) is observed. A band at 650 cm^−1^ is correlated with the vibrations of gypsum. A band at 876 cm^−1^ is attributed to unreacted slag. Meanwhile, at 1435 cm^−1^, a band attributed to C-S-H gel is also observed. The O-C-O vibrations related to calcium carbonate correspond to the band identified at 1480 cm^−1^. At 1635 cm^−1^, a band is associated with O-H bonds. The broad band at 3500 cm^−1^ is linked to the H-OH bonds of the reaction water [88].

Figure 12b presents the spectra for the 60S40MFA10C pastes. The same bands observed in Figure 12a are present, except for the bands at 700 cm^−1^ and 460 cm^−1^, which correspond to the Si-O bonds in the anhydrous ash. A prominent band at 876 cm^−1^ is attributed to unhydrated slag, associated with the asymmetric stretching of AlO_4_. Additionally, a band at 1018 cm^−1^ corresponds to the asymmetric stretching of the Si-O-T bonds in the SiO_4_^2−^ structure. An associated band characteristic of the C-S-H gel can be observed at 1435 cm^−1^. At 2530 cm^−1^, the band is correlated with limestone (CO_3_^2−^). A band at 2919 cm^−1^ corresponds to the vibrational mode of hydrotalcite [87]. Meanwhile, at 3500 cm^−1^, a band is also observed, which is attributed to the H-O bonds of portlandite-Ca(OH)_2_ [9,89]. Also, H-O bonds from Ca(OH)_2_ are observed at 980 cm^−1^ [90].

Table 3 describes the frequencies and bond types related to the hydration gels found in the FTIR analysis of granulated blast-furnace slag and ground fly ash.

The FTIR analysis indicates the formation of reaction products resulting from the activation of ground fly ash and blast-furnace slag. Changes become noticeable around 900 cm^−1^, with an increasing degree of polymerization as the reaction progresses. In this paste, the calcium–silicate–hydrate (C-S-H) incorporates AlO_4_^−^ and Na^+^ as substitutes for SiO_4_^4−^ [91].

### 3.6. Calorimetry (Heat Flow)

**Figure 13:**
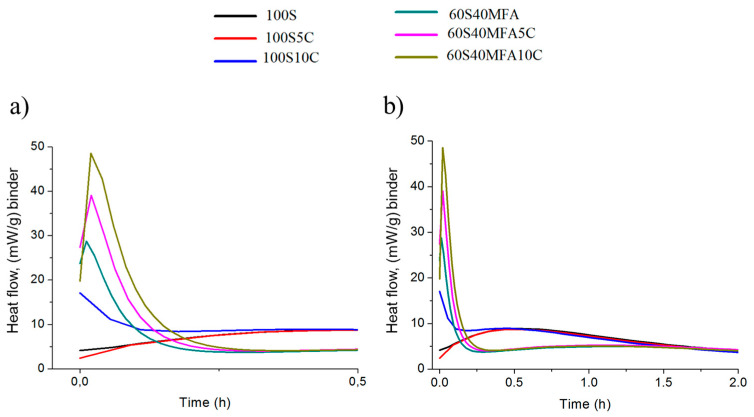
Heat flow of mixtures: (**a**) 0.5 h and (**b**) time scale of 2 h.

The hydration curves have distinct induction, acceleration, deceleration, and steady- state diffusion stages [90]. Figure 13 shows the heat flow during the hydration of the experimental pastes. The calorimetric response in Figure 13a shows two peaks: an early dissolution and a later acceleration peak in the 100S, 100S5C, and 100S10C pastes. The very early narrow peak within the first few seconds of mixing, shown in Figure 13a, corresponds to the wetting and dissolution of slag particles [14,91]. The dormant period after this peak is followed by an acceleration peak that is smaller in magnitude. The induction period in the alkali-activated 100S, 100S5C, and 100S10C pastes is prolonged [91]. This phenomenon is because of the time required for the ionic species in the solution to reach a critical concentration to form reaction products. The alkali-activated 100S, 100S5C, and 100S10C pastes have broader acceleration peaks, suggesting better activation, i.e., an enhanced reaction of slag after the dormant period, as can be observed from the larger amplitude of the acceleration peak. Therefore, the 100S, 100S5C, and 100S10C pastes developed their initial reaction and acceleration during the first seconds of the exothermic alkaline hydration reactions.

The calorimetric responses of 60S40MFA, 60S40MFA5C, and 60S40MFA10C demonstrate broader, low-intensity acceleration peaks occurring in the first half an hour in addition to the initial intense peaks. The appearance of the low-intensity acceleration peaks in the 60S40MFA, 60S40MFA5C, and 60S40MFA10C pastes can be attributed to the formation of C–S–H gel in a diluted Ca-containing system under the influence of an alkaline activator.

The initial intense peak behavior observed for the 60S40MFA (27 mW/g), 60S40MFA5C (39 mW/g), and 60S40MFA10C (48 mW/g) pastes evaluated here can be attributed to a combination of particle wetting, the dissolution of slag, and the formation of aluminosilicate complexes that incorporate alkali and calcium ions [91,92]. It is also possible that the rapid dissolution and reaction product precipitation, along with the external mixing adopted for the calorimetric studies, could have rendered the distinct pre-induction and induction periods undetected [91]. For the 60S40MFA, 60S40MFA5C, and 60S40MFA10C pastes, the observed peaks are steeper and prolonged. This fact is due to the acceleration of the initial reaction facilitated by high amounts of [SiO_4_]^4−^ ions.

From the 2 h time graph (Figure 13b), it is observed that the 60S40MFA, 60S40MFA5C, and 60S40MFA10C pastes entered the deceleration period in the following minutes until reaching 2 h, exhibiting a slow reaction process. The 100S, 100S5C, and 100S10C pastes developed heat flow reactions from 25 to 90 min of approximately 10 mW/g heat. This fact is possibly associated with the slag reacting with the limestone added.

**Figure 14:**
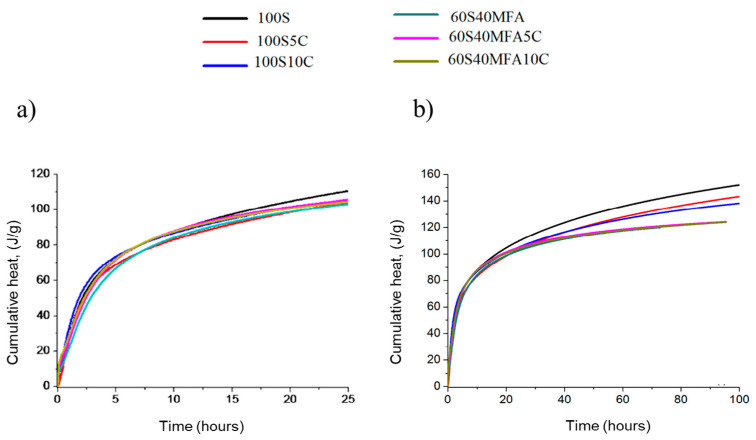
Heat flow of all mixtures with limestone additions. (**a**) Time scale of 100 h and (**b**) 25 h.

Figure 14 shows the cumulative heat release of the activated slag and fly ash/slag pastes after 100 h of reaction. The initial rising portion shows the heat-release contribution due to the wetting and dissolution of the slag, and the second rising portion depicts the heat-release contribution due to the acceleration phase of the reaction. The induction period is represented by the relatively flatter region in the cumulative heat-release curve between the initial dissolution rise and the second acceleration rise. The long dormant period for the activated slag and fly ash/slag pastes results in a pronounced plateau in the cumulative heat curve. The 100S paste developed a higher heat of hydration (105 J/g) than the rest of the pastes, which reached 85 J/g (see Figure 14a).

The limestone addition helped to decrease the heat. Pastes with higher slag content (100S, 100S5C, and 100S10C) developed a higher heat of hydration. Meanwhile, the 60S40MFA pastes showed gradual behavior with almost no variation (see Figure 14b) during the analysis from 20 h (100 J/g) to 100 h (110 J/g).

### 3.7. Summary Discussion

The analysis of alkali-activated systems revealed critical insights into the chemical and mechanical properties of slag-based and slag/fly ash blended pastes, with and without limestone addition. The reaction between calcium carbonate (CaCO_3_) and sodium hydroxide (NaOH) produces calcium hydroxide (Ca(OH)_2_), commonly identified as portlandite through XRD, FTIR, and SEM analyses. XRD revealed portlandite peaks at 2θ angles of 16°, 29°, and 34°, while FTIR analysis detected characteristic bands at 1031 cm^−1^ and 3640 cm^−1^. The SEM analysis revealed hexagonal morphologies of portlandite particles, which are crucial in alkali-activated cements (AACs). These particles release calcium ions that promote the formation of calcium–silicate–hydrate (C-S-H) and calcium–aluminosilicate–hydrate (C-A-S-H) gels. These gels are vital for enhancing early strength, long-term durability, and resistance to environmental stresses.

XRD analysis confirmed the presence of calcite, quartz, mullite, and merwinite in the 100S, 100S5C, and 100S10C pastes. In systems with limestone, tobermorite (a type of C-S-H gel) and hydrotalcite phases were detected, although no new crystalline phases formed. The XRD analysis of the 60S40MFA, 60S40MFA5C, and 60S40MFA10C pastes confirmed the presence of calcite, quartz, mullite, merwinite, tobermorite, and hydrotalcite.

FTIR analysis identified C-S-H, N-A-S-H, and C-A-S-H gels with unique vibrational bands corresponding to their respective structures. C-S-H gels were associated with bands at 900–1100 cm^−1^ (Si-O stretching), 460–500 cm^−1^ (Si-O bending), and carbonate-related bands at 1430–1450 cm^−1^. N-A-S-H gels showed Si-O-Al and Si-O-Si asymmetric stretching bands near 950–1000 cm^−1^. Hybrid (N, C)-A-S-H gels often exhibit bands at 950–1000 cm^−1^ for the Si-O-T stretching band, with additional bands near 1435 cm^−1^ due to carbonate vibrations reflecting calcium integration.

The formation of these gels occurs in varying ratios, depending on the paste composition and calcium content. Hybrid gels provide rigidity and flexibility, making them particularly beneficial for alkali-activated cement (AAC) systems.

Heat flow analysis showed distinct hydration profiles for the slag and slag/fly ash blends. Slag pastes exhibited a sharp acceleration peak after the dormant phase, indicating rapid activation and vigorous early reactions that transition to gel formation. In slag/fly ash blends, hydration was slower, with a broader and less intense secondary peak reflecting gel formation in calcium-deficient environments.

Microstructurally, slag pastes with limestone addition (e.g., 100S10C) demonstrated dense, compact matrices. Ca/Si ratios between 1.0 and 4.7 favored C-S-H gel formation. Hybrid (N, C)-A-S-H gels were observed in zones with intermediate Ca/Si ratios (1.0–1.3), indicating optimal conditions for matrix integration. Mg/Al ratios between 1.0 and 1.38 suggested the formation of hydrotalcite phases in specific regions.

In slag/fly ash blends, limestone promotes diverse gel formations, including C-S-H, N-A-S-H, and hybrid (N, C)-A-S-H gels. Ca/Si ratios from 1 to 6.3 promoted C-S-H gel formation. Lower Ca/Si ratios (0.5–1.0) and Si/Al ratios ≥ 2 promoted N-A-S-H gel development. Meanwhile, (N, C)-A-S-H gels were detected in regions with Ca/Si ratios between 1.0 and 1.3, highlighting the matrix’s capacity for multiple gel formations.

In terms of the slag pastes, the 100S paste achieved the highest compressive strength (48.87 MPa at 28 days), attributed to the dense C-S-H and C-A-S-H gel network. Moreover, while limestone addition (100S5C and 100S10C) improved early strength (up to a 9.31% increase at 7 days), it slightly reduced the 28-day strength (maximum 46.68 MPa for 100S5C).

Slag/fly ash blends showed lower compressive strengths, with a peak of 42.24 MPa for 60S40MFA. This reduction is linked to the lower reactivity of fly ash at ambient temperatures and insufficient alkaline liquid due to the blend’s increased specific surface area. Limestone addition marginally improved early strength but had minimal impact at 28 days.

The addition of limestone improved the flexural strength of the pastes. The highest strength values observed were 8.35 MPa for the 100S10C paste and 5.44 MPa for the 60S40MFA10C paste. Hybrid (N, C)-A-S-H gel formation in the slag/fly ash blends contributed to enhanced flexibility, providing the matrix with higher resistance against flexural stresses.

All pastes exceeded the ASTM C 150-95 minimum compressive strength of 20 MPa for OPC 20R. Limestone addition enhanced gel diversity and mechanical performance, particularly in terms of flexural strength, while maintaining adequate compressive strength. The hybrid gel structures, especially (N, C)-A-S-H, contributed to durability, carbonation resistance, and flexibility in the AACs and geopolymers, offering significant potential for diverse applications.

## 4. Conclusions

This study investigates the effects of μ-limestone addition on slag and binary slag/ground fly ash alkaline-activated binders, focusing on Ca^2+^ leaching, its impact on reaction kinetics, and mechanical properties. The results highlight key aspects of interactions, hydration mechanisms, and microstructural development in these systems.

μ-Limestone remains inert in neutral conditions but reacts in alkaline environments. XRD, FTIR, and SEM analyses confirm the interaction between μ-limestone (CaCO_3_) and sodium hydroxide (NaOH), forming portlandite (Ca(OH)_2_). Portlandite is identified through its XRD peaks (16°, 29°, and 34° 2θ) and FTIR bands (1031 cm^−1^ and 3640 cm^−1^), appearing as hexagonal particles under SEM analysis. The dissolution of μ-limestone in NaOH releases calcium ions that react with N-A-S-H gels derived from fly ash activation, leading to hybrid (N, C)-A-S-H gel formation.

The XRD analysis of slag/fly ash blends confirmed the presence of key phases such as tobermorite (C-S-H gel) and hydrotalcite, highlighting limestone’s role in modulating both short- and long-term hydration without introducing new crystalline phases. The FTIR analysis identified distinct vibrational bands for the C-S-H, N-A-S-H, and C-A-S-H gels, which evolve depending on calcium availability and gel composition. Hybrid gels exhibit characteristics of both N-A-S-H and C-A-S-H phases, influenced by calcium carbonate dissolution.

The microstructural analysis revealed significant insights. The pure slag paste with 10% limestone (100S10C) exhibited a dense and uniform structure, with Ca/Si ratios ranging from 1.5 to 4.7, conducive to robust C-S-H gel formation. (N, C)-A-S-H hybrid gels appeared in regions with Ca/Si ratios from 1.0 to 1.3, producing a durable and well-integrated matrix. Mg/Al ratios between 1 and 1.38 indicated hydrotalcite phase formation within the structure.

In the slag/fly ash blends, limestone promotes diverse gel formations, including C-S-H, N-A-S-H, and hybrid (N, C)-A-S-H gels. Ca/Si ratios from 1 to 6.3 promoted C-S-H gel formation. Lower Ca/Si ratios (0.5–1.0) and higher Si/Al ratios (≥2) encourage N-A-S-H gel formation. Meanwhile, (N, C)-A-S-H gels were detected in regions with Ca/Si ratios between 1.0 and 1.3, highlighting the matrix’s capacity for multiple gel formations.

Compressive strength at 28 days reached 48.87 MPa for the pure slag pastes (100S), attributed to dense C-S-H and C-A-S-H gel formation, which minimized porosity and enhanced matrix densification. The limestone addition did not significantly improve early-age strength in the pure slag pastes, but by 7 days, minor increases (9.31% and 2.45%) were observed for 100S5C and 100S10C, respectively. However, a slight reduction in 28-day strength was noted, with the highest limestone-containing paste strength at 46.68 MPa (100S5C).

For slag/fly ash blends, the highest strength at 28 days was 42.24 MPa (60S40MFA), which was lower than the pure slag systems due to fly ash’s lower reactivity at ambient conditions. The replacement of slag with fly ash increased paste surface area, reducing available alkaline liquid and limiting gel product formation. Limestone-containing slag/fly ash pastes achieved a maximum strength of 40.47 MPa (60S40MFA5C). The flexural strength results showed a positive influence of limestone, with the highest value of 8.35 MPa observed in 100S10C.

The heat flow analysis revealed a prominent acceleration peak for the alkali-activated slag pastes, indicating effective activation and rapid transition from the induction to acceleration phases. The slag/fly ash blends exhibited a broader and lower-intensity peak associated with C-S-H formation in calcium-deficient environments, underscoring the activator’s role in early hydration.

These findings demonstrate that μ-limestone-modified AACs meet mechanical strength standards (≥20 MPa per ASTM C150-95), making them suitable for application in mortars, industrial floors, masonry, and soil stabilization.

## Figures and Tables

**Table 1:** Chemical analysis of the raw materials (wt%) by XRF.

Raw Material	MgO	Al_2_O_3_	SiO_2_	SO_3_	K_2_O	CaO	TiO_2_	MnO	Fe_2_O_3_	Loss on Ignition (LOI)
Slag (S)	9.57	9.71	32.16	3.24	0.4264	42.2494	1.6226	0.1414	0.4122	2.5
Ground fly ash (MFA)	0.635	26.72	61.94	1.035	3.679	2.804	0.93	0.01	4.2475	2.7
Limestone (LSP)	1.06	0.42	1.6	0	0.01	97.04	0	0	0.01	-

**Table 2:** Mixture proportions (wt%).

Code	Binders (wt%)			NaOH Activator	L/S Ratio
	Slag	MFA	LSP		
100S	100	-	-	4M	0.30
100S5C	100	-	5	4M	0.30
100S10C	100	-	10	4M	0.30
60S40MFA	60	40	-	4M	0.35
60S40MFA5C	60	40	5	4M	0.35
60S40MFA10C	60	40	10	4M	0.35

**Table 3:** Characteristic band frequencies of the types of bonds according to FTIR.

Blast-Furnace Slag (S)		Ground Fly Ash (MFA)	
Frequency (cm^−1^)	Bond Type	Frequency (cm^−1^)	Bond Type
3500 cm^−1^	(O-H)	1635 cm^−1^	O-H-O (H_2_O)
1635 cm^−1^	(H-OH^−^)	-	-
1470 cm^−1^	[CO_3_]^2−^	-	-
1440 cm^−1^	[CO_3_]^2−^	1100 cm^−1^	T-O (T:Si or Al)
876 cm^−1^	[CO_3_]^2−^	789 cm^−1^	Si-O quartz_(asymmetric stress)_
712 cm^−1^	[CO_3_]^2−^	764 cm^−1^	Si-O quartz_(asymmetric stress)_
964 cm^−1^	(Si-O)	700 cm^−1^	Si-O
500 cm^−1^	(O-Si-O)		
460 cm^−1^		556 cm^−1^	T-O (mullite)
1050 cm^−1^	Al-O	460 cm^−1^	O-Si-O (TO_4 tetrahedra_)

## Data Availability

Data are contained within the article.
